# Wnt Signaling Pathways in Keratinocyte Carcinomas

**DOI:** 10.3390/cancers11091216

**Published:** 2019-08-21

**Authors:** Christopher M. R. Lang, Chim Kei Chan, Anthony Veltri, Wen-Hui Lien

**Affiliations:** de Duve Institute, Université catholique de Louvain, Brussels 1200, Belgium

**Keywords:** Wnt signaling, non-melanoma skin cancer, basal cell carcinoma, squamous cell carcinoma

## Abstract

The skin functions as a barrier between the organism and the surrounding environment. Direct exposure to external stimuli and the accumulation of genetic mutations may lead to abnormal cell growth, irreversible tissue damage and potentially favor skin malignancy. Skin homeostasis is coordinated by an intricate signaling network, and its dysregulation has been implicated in the development of skin cancers. Wnt signaling is one such regulatory pathway orchestrating skin development, homeostasis, and stem cell activation. Aberrant regulation of Wnt signaling cascades not only gives rise to tumor initiation, progression and invasion, but also maintains cancer stem cells which contribute to tumor recurrence. In this review, we summarize recent studies highlighting functional evidence of Wnt-related oncology in keratinocyte carcinomas, as well as discussing preclinical and clinical approaches that target oncogenic Wnt signaling to treat cancers. Our review provides valuable insight into the significance of Wnt signaling for future interventions against keratinocyte carcinomas.

## 1. Introduction

The skin is the largest organ and functions as a protective barrier for the host to prevent fluid loss and regulate body temperature. It also harbors vasculature and sensory organs that transduce changes in temperature and pressure. The skin is composed of two major layers, the epidermis and dermis, forming the outer and inner layers, respectively. The epidermis includes the stratified epithelium, referred to as the interfollicular epidermis (IFE), and skin appendages, including the hair follicle, sebaceous gland, and sweat gland. The IFE and the hair follicle undergo regeneration throughout life. Constant exposure to mutagens, i.e., ultraviolet (UV) light and chemicals, can induce genetic mutations and hyperproliferation of epidermal cells, both of which eventually contribute to the formation of skin cancer. Two major types of non-melanoma skin cancer (NMSC) commonly found in patients are basal cell carcinoma (BCC) and squamous cell carcinoma (SCC), which together account for more than 95% of total NMSC [1]. As BCC and SCC share lineage with epidermal keratinocytes, they are specifically referred as keratinocyte carcinomas [2,3]. Although the risk of death from keratinocyte carcinomas is moderate, it increases considerably if patients are immunocompromised. Thus, identifying the drivers that induce skin tumors and understanding the molecular mechanisms responsible for tumor progression and maintenance is critical for biomarker discovery in diagnosis, prognosis, and therapy monitoring. 

Several signaling pathways that have been shown to play vital roles in development of the skin epithelium are also implicated in the progression of keratinocyte carcinoma, including hedgehog (Hh), transforming growth factor β (TGFβ), mitogen-activated protein kinase (MAPK/ERK) and Wnt signaling [4,5,6,7]. Among them, Wnt signaling is of particularly interest due to its complexity in intracellular signaling cascades triggered by differential Wnt ligand-receptor combinations. Divergent roles of Wnt signaling have been discovered in multiple mammalian tissues during development and homeostasis of adult tissues. Canonical Wnt/β-catenin signaling is a major regulatory pathway that governs developmental processes as well as regulating maintenance and differentiation of adult stem cells (SCs) [8,9,10]. In this review, we summarize recent studies where the role of Wnt signaling in regulating tumor initiation and progression of keratinocyte carcinoma has been identified.

## 2. Wnt Signaling Pathways

Wnt signaling pathways govern a multitude of cellular functions including cell proliferation, differentiation, fate specification, migration and polarity [11,12,13]. As such, Wnt signaling regulation is decisive and its aberrant activity can lead to developmental defects or various pathogenesis, including cancer [10,14]. 

**Figure 1:**
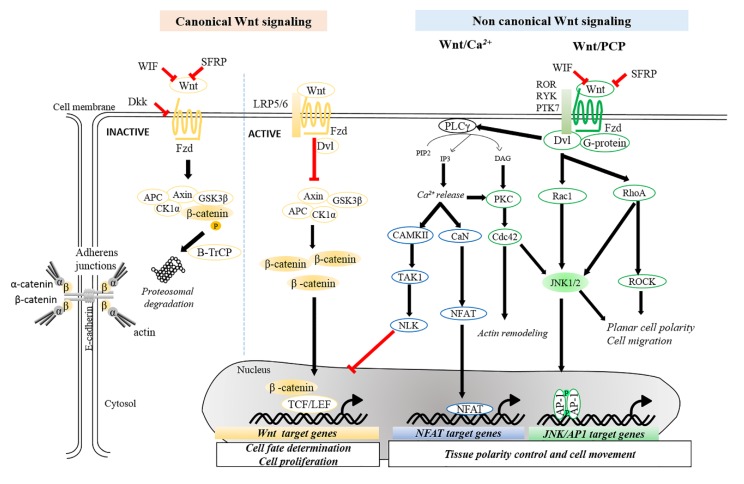
Overview of Wnt signaling pathways. This schematic diagram illustrates simplified canonical (β-catenin-dependent) and non-canonical (β-catenin-independent) Wnt signaling pathways. In the absence of Wnt, β-catenin is targeted by a destructive complex, composed of Axin, CK1α, APC, and GSK3β, for degradation. Upon Wnt binding to the receptor, recruited Dvl inhibits the degradation complex, which in turn stabilizes β-catenin. Stabilized β-catenin accumulates in the cytoplasm and then enters the nucleus where it acts as a transcriptional co-activator for TCF/LEF transcription factors to activate Wnt target genes. The transcriptional activation mediated by β-catenin can be suppressed by NLK, which is activated through non-canonical Wnt pathways. Non-canonical Wnt signaling is divided into Wnt/Ca^2+^ and Wnt/PCP pathways. Wnt/Ca^2+^ signaling is characterized by the release of intracellular Ca^2+^ via activation of PLCγ converting PIP2 into IP3 and DAG, thus activating CaN, CaMKII or PKC. For Wnt/PCP pathways, Wnt ligand-receptor interaction activates small GTPases Rho or Rac, allowing cytoskeletal reorganization and modulating downstream JNK signaling. AP-1, activating protein-1; APC, adenomatous polyposis coli; CaMKII, Ca^2+^/calmodulin-dependent protein kinase II; CaN, calcineurin; CK1α, casein kinase 1α; DAG, diacylglycerol; Dkk, dickkopf proteins; Dvl, dishevelled; Fzd, Frizzled; GSK3β, glycogen synthase kinase 3β; IP3, inositol 1,4,5-triphosphate; JNK, c-Jun N-terminal kinase; TCF/LEF, T-cell factor/lymphoid-enhancing factor; LRP5/6, lipoprotein receptor-related protein 5/6; NFAT, nuclear factor of activated T-cells; NLK, nemo-like kinase; PCP, planar cell polarity; PIP2, phosphatidylinositol 4,5-bisphosphate; PKC, protein kinase C; PLCγ, phospholipase C γ; PTK7, tyrosine-protein kinase-like 7; ROCK, Rho-associated kinase; ROR, receptor tyrosine kinase-like orphan receptor; RYK, receptor-like tyrosine kinase; SFRP, secreted frizzled-related proteins; WIF, Wnt inhibitory factor.

Wnt signal transduction is initiated upon the binding of Wnt ligands to cell surface receptors of the Frizzled (Fzd) family. Upon binding, Fzd can associate with other co-receptors, such as low-density lipoprotein-related protein (LRP5/6) or tyrosine kinase receptors (PTK7, ROR, RYK), to activate Dishevelled (Dvl) and then trigger diverse signaling cascades [15,16,17,18]. Wnt signaling is generally classified into β-catenin-dependent canonical (referred as Wnt/β-catenin signaling) and β-catenin-independent non-canonical pathways (Figure 1).

The activation of canonical Wnt signaling is characterized by β-catenin-dependent transcriptional activity. In the absence of Wnt ligand, cytosolic β-catenin is captured and phosphorylated by the destruction complex, composed of Axin, adenomatous polyposis coli (APC), casein kinase 1 (CK1), and glycogen synthase kinase 3β (GSK3β) [19,20,21]. Captured β-catenin is subsequently bound by β-transducin repeat-containing protein (β-TrCP), which mediates ubiquitylation and proteasomal degradation of β-catenin. Upon Wnt-receptor interaction, the destruction complex is inhibited, which in turn stabilizes cytosolic β-catenin. Stabilized β-catenin enters the nucleus, and then interacts with members of the T-cell factor (TCF)/lymphoid enhancer factor (LEF) family to trigger the transcription of Wnt target genes, e.g., *Axin2*, c-*Myc*, and *Ccnd1* [22,23]. 

Non-canonical Wnt signaling transduces signals independent of β-catenin, and can be divided into Wnt/Calcium (Ca^2+^) and Wnt/planar cell polarity (PCP) pathways [24,25,26]. In the Wnt/Ca^2+^ pathway, Wnt-Fzd interaction leads to the activation of phospholipase C and increases the concentrations of inositol 1,4,5-triphosphate (IP3) and 1,2 diacylglycerol (DAG). IP3 interacts with intracellular calcium channels to release Ca^2+^ ions, leading to the activation of calcium-dependent kinases, such as protein kinase C (PKC), Ca^2+^-calmodulin dependent kinase II (CAMKII), or Ca^2+^-dependent phosphatase calcineurin (CaN) [27,28,29]. PKC has been shown to activate the small GTPase Cdc42 [30] while CAMKII phosphorylates TGFβ-activated kinase 1 (TAK1), which in turn induces Nemo-like kinase (NLK) activation, which inhibits the transcriptional activity of Wnt/β-catenin signaling [31]. In parallel, CaN dephosphorylates nuclear factor of activated T-cells (NFAT) family proteins and causes their nuclear translocation, allowing transcriptional regulation of their target genes [32]. Activation of the Wnt/Ca^2+^ pathway triggers a wide-range of cellular processes, including actin cytoskeleton remodeling and cell motility [33]. For the Wnt/PCP pathway, the binding of Wnt ligands to their receptors activates Rho-family small GTPases, including RhoA and Rac, and their downstream effectors, Rho-associated protein kinase (ROCK), the actin-binding protein Filamin A and c-Jun N-terminal protein kinase (JNK) [34,35]. Among of these, activated JNK further triggers transcriptional activation of activating protein-1 (AP-1) family of transcription factors [36]. As AP-1 proteins also act as downstream effectors of several signaling pathways, e.g., RAS pathway [37,38], the cross-interaction of Wnt signaling with other pathways may occur in a context-dependent manner. 

The transduction of Wnt signals depends not only on which ligand is present, but also on which receptor(s) and cognate receptor(s) are expressed in the cell. As such, a single Wnt protein can trigger a combination of multiple signaling cascades that might work together as a dynamic signaling network [39].

## 3. Wnt Signaling in Skin Homeostasis and Regeneration

The adult skin epidermis is composed of the IFE, hair follicles, sebaceous glands and eccrine sweat glands. Cellular processes including homeostatic maintenance and post-damage regeneration are ensured by the multipotent epidermal SC populations located in both the basal layer of IFE and in the hair follicle [40].

The IFE is continuously being regenerated by cells within the basal layer, which proliferate and give rise to cells that migrate outwards and differentiate into suprabasal keratinocytes, and then terminally differentiate into cornified envelope cells. The control of basal cell proliferation within the IFE is tightly regulated by Wnt/β-catenin signaling [41,42]. Absence of Wnt/β-catenin signaling in the embryonic IFE results in hyperproliferation, which is caused either by degeneration of HFs or by other intertwined factors, such as impairment of skin barrier integrity and inflammation [41,43]. By contrast, when Wnt/β-catenin signaling is suppressed in basal cells of non-hairy epidermis, the epidermis exhibits severe hypoproliferation [42,44].

In mammalian skin, mature HFs undergo regeneration by progressing through cyclical phases of growth (anagen), degeneration (catagen), and rest (telogen). This long-lasting regeneration is fueled by hair follicle stem cells (HFSCs). The activation of HFSCs is tuned by a balance of bone morphogenetic proteins (BMP) and Wnt signals coming from their niche cells [45]. During telogen, HFSCs remain quiescent as they reside in the niche where inhibitory signals, e.g., BMP6 and fibroblast growth factors 18 (FGF18), and Wnt antagonists, e.g., secreted frizzled receptor protein 1 (SFRP1), Wnt inhibitory factor 1 (WIF1), and Dickkopf-related protein 3 (Dkk3), are present at high levels [46,47]. At the end of telogen, BMP signals from the niche are reduced, which allows HFSCs to transduce Wnt/β-catenin signaling and thereby promote anagen entry [48]. The importance of Wnt/β-catenin signaling in HF regeneration is supported by genetic studies showing that transient ectopic activation of β-catenin in adult epidermis is sufficient to induce new hair growth [49], and deletion of β-catenin in HFSCs results in impairment of HF regeneration [44,50,51]. 

Beyond the role of Wnt signaling in the normal regeneration cycle of the hair follicle, Wnt signaling also plays a role in the acute response to injury. Upon injury, the adult skin epidermis undergoes a wound healing process which occurs in four overlapping phases: disruption of homeostasis, inflammation, re-epithelialization, and tissue remodeling [52]. Gene expression profiling of wounds and carcinomas indicates significant similarities between the tumor development and wound healing processes. Indeed, a malignant tumor is considered as an overhealing wound, in which permanent tissue injury induces chronic inflammation causing the development of cancer [53,54]. It has been shown that Wnt signaling participates in the process of wound healing from the control of inflammation to mobilization of SCs within the wound site [55,56].

In summary, epidermal SCs undergo self-renewal and differentiation to regenerate the skin epidermis during homeostasis and upon injury. A growing body of evidence shows that tumor growth is driven by a population of tumor cells presenting SC-like characteristics, such as self-renewal and slow-cycling properties [57]. Thus, SC populations in different skin compartments would have served as origins of different skin malignancies. This rationale will be elaborated further in the next section. 

## 4. Wnt Signaling in Keratinocyte Carcinomas

While genetic mutations are major factors for the development of keratinocyte carcinoma, disrupted signaling pathways have emerged as necessary cofactors. Disruption of HF homeostasis may lead to hair loss disorders, such as alopecia universalis, or uncontrolled HF proliferation, which may cause follicle-based tumors [58]. Notably, aberrant activation of Wnt/β-catenin signaling is considered as one of the main driving elements causing developmental defects and tumorigenesis [14]. The constitutive expression of stabilized β-catenin in skin epidermis (K14-∆Nβ-catenin) causes the development of pilomatricomas or trichofolliculomas [59,60]. In contrast, depletion of β-catenin in carcinogen-induced SCCs results in tumor regression [61], indicating the essential role of β-catenin-dependent signaling in tumorigenesis. Genome-wide RNA-interference (RNAi) screening in the developing skin epidermis has reinforced the notion that β-catenin contributes to oncogenic growth [62]. Furthermore, in addition to the TCF/LEF transcription factor, nuclear β-catenin can also bind to the vitamin D receptor (VDR), which was shown to play a role in HF maintenance [63,64]. Inhibition of β-catenin-dependent transcriptional activity by overexpressing N-terminally truncated Lef1 in mouse epidermis (K14-∆NLef1) leads to the development of sebaceous gland (SG) tumors [65,66]. Interestingly, activation of β-catenin signaling (K14-∆Nβ-cateninER) in the absence of VDR causes development of tumors resembling BCCs [67]. These findings suggest that skin tumor types are specified by the interaction between β-catenin and its transcriptional effectors. Moreover, oncogenic activation of β-catenin in different epidermal SC populations results in distinct tumor types [68], implicating skin tumor heterogeneity. In addition to canonical Wnt signaling, non-canonical Wnt signaling is also implicated in epithelial-to-mesenchymal transition (EMT), a process involved in tumor metastasis and chemo-repulsion [69,70]. In the following sections, we focus on BCC and SCC, and discuss the implications of the Wnt signaling pathways in tumor formation and progression of these tumors.

### 4.1. Basal Cell Carcinoma (BCC)

BCC is the most common skin tumor in humans. The main etiological factors provoking the development of BCCs are UV radiation, ionizing radiation, arsenic exposure, as well as traumatic injury or burn [71,72]. Other factors, such as wounding, can increase the risk of BCC development and malignancy in humans and mice [73,74]. It is noteworthy that BCCs are readily treated by means of various surgical methods at an early stage [75], and in some exceptional cases, BCC is reported to undergo self-regression [76,77,78]. However, once these lesions progress from BCC in situ to an advanced state, they are no longer amenable to surgery or radiation therapy. In even more rare cases, the tumor cells spread to distant sites (metastatic BCC). Although metastatic rate (<0.1%) and mortality caused by BCC is low, it may create substantial damage to skin tissue or morbidity if neglected for prolonged periods [79,80,81].

BCCs can be clinically and histologically categorized into several encompassing nodular, micronodular, superficial, infiltrative, morpheiform, and mixture variants. Thus, BCC is generally characterized based on the structure of tumor cells similar to the basal cells of the normal epidermis, however, their molecular characteristics are more related to embryonic hair follicle progenitors [82]. Recently, a new molecular classification was introduced apart from clinical and histopathological classifications. Based on the genomic profiling, BCCs are divided into three subtypes: 1) classical BCCs, which are closely associated with the Wnt and Hh signaling pathways; 2) normal-like BCCs, notably displaying an active fatty acid metabolism; and lastly 3) SCC-like BCCs, relying on immune-response and oxidative stress-related genes [83]. Here, we mainly focus on classical BCCs.

**Figure 2:**
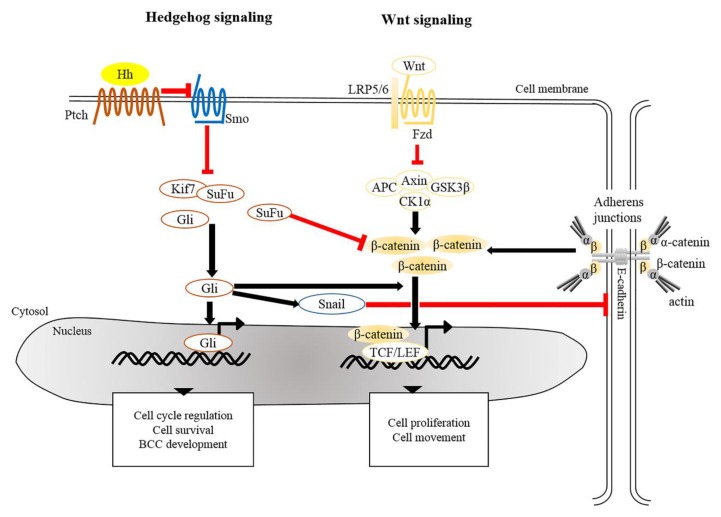
Crosstalk of signaling pathways in the pathogenesis of BCC. The main driver of BCC development is the dysregulation of Hedgehog signaling and Wnt/β-catenin signaling whereby the *PTCH*, *SMO*, *SUFU*, and *CTNNB1* (a gene encoding for β-catenin) are frequently mutated in human BCCs. Mutated Ptch loses its grip on Smo that subsequently leads to its activation. Aberrant activation of Smo releases inhibition of Gli from SuFu and Kif7, thus allowing nuclear translocation of Gli. Aberrant Gli activity induces the expression of genes that regulate the cell cycle, cell survival and development of BCC. In addition, Gli induces Snail leading to the inhibition of E-cadherin, which in turn results in the accumulation of free β-catenin and its translocation to the nucleus. Alternatively, inactivation of SuFu and Kif7 leads to the accumulation and nuclear translocation of stabilized β-catenin which in turn facilitates BCC genesis. BCC, basal cell carcinoma; Gli, Glioma associated oncogenic homolog; Kif7, kinesin family member 7; Ptch, Patched; Smo, Smoothened; SuFu, Suppressor of Fused.

There is extensive evidence that the origin of BCC pathogenesis is predominantly triggered by dysregulation of the Hh pathway [84,85]. This could be attributable to aberrant genetic alterations that inactivate Patched 1 (PTCH1) or Suppressor of Fused (SUFU), constitutively activate Smoothened (SMO), or lead to overexpression of Glioma associated oncogene homolog 1 (GLI1) [4,72,86]. In the absence of Hh, Ptch acts to prevent activity of Smo. When Hh binds to and inhibits Ptch, Smo is activated to release inhibition of Gli by SuFu and kinesin family member 7 (Kif7), thus allowing Gli to enter the nucleus and initiate transcriptional activation of genes that regulate cell survival, cell cycle regulation and angiogenesis (Figure 2) [87,88,89]. Uncontrollable activation of Hh signaling prominently promotes tumorigenesis in sporadic and inherited BCCs. Based on recent genomic analysis, loss of *PTCH1* and gain of *SMO* were described as causative mutant Hh pathway genes, accounting for 90% of human BCCs [72,90]. Mouse models of BCC genesis mainly rely on the repression of Patch1 or overexpression of Gli1/2. For example, mice overexpressing Gli1 in skin epidermis (K5-Gli1) develop several types of skin tumors, primarily hair follicle-derived tumors and BCCs [91], whereas Gli2 overexpression (K5-Gli2) only causes the formation of BCCs [92]. Thus, alterations in the expression of Hh signaling components may lead to the development of different tumor types. Moreover, the original cell populations in skin epidermis expressing excess Hh signaling also determine the phenotype of developed BCCs. For instance, over-activation of Gli2 in IFE gives rise to superficial BCC-like tumors, whereas HFSCs overexpressing Gli2 develop nodular BCC-like tumors [93]. Apart from IFE and HFs, innervated progenitors within mechanosensory niches were shown to be another plausible cell population that contributes to the development of BCCs [94]. 

The development and progression of tumors are orchestrated by a network of intricate signaling pathways. In addition to Hh signaling, ample scientific evidence indicates that the Wnt/β-catenin signaling pathway participates in tumorigenesis of BCCs. Of note, constitutive expression of Wnt mediators, e.g., Wnt1, 2, 5A, 11, 13, and 16 and β-catenin, facilitates the progression of BCCs [95,96]. As reported previously, nearly 30% of human BCC samples exhibited an accumulation of β-catenin in the nucleus [97,98], and nuclear β-catenin in BCCs mainly resides at the tumor periphery [99]. In addition, BCCs which exhibit nuclear β-catenin display significantly higher proliferation rates [100]. Along this line, 25% of BCC samples contain the missense mutations in the third exon of the β-catenin gene [101], which is considered as a notable characteristic of hair follicle-related skin carcinoma [60,102]. Mutations in exon 3 of *CTNNB1* (the gene encoding for β-catenin)*,* particularly at Ser 33, 37 and Thr 41, perturb the phosphorylation sites for GSK3β, which leads to stabilization of β-catenin and in turn elevates Wnt/β-catenin signaling in favor the event of tumorigenesis [102,103]. As mentioned earlier, activation of β-catenin in the absence of VDR in mouse epidermis results in the development of undifferentiated tumors resembling BCCs [67]. Moreover, transcriptional profiling of adult epidermis expressing constitutively active SmoM2 shows that adult tumor-initiating cells are reprogramed into an embryonic hair follicle progenitor-like fate, in which Wnt/β-catenin signaling is highly activated. Depletion of β-catenin in adult epidermis expressing SmoM2 prevents embryonic reprograming and skin tumorigenesis [82]. Indeed, during skin development, β-catenin-dependent signaling directs the embryonic ectoderm to a HF-like fate [104,105], and over-activated β-catenin induces de novo HFs in adult epidermis [59]. Hence, β-catenin represents as a cardinal target in BCC arising from a HF-related origin.

Crosstalk between Hh and Wnt signaling pathways have been recently implicated in the pathogenesis of BCCs. Several genomic studies have indicated that genes encoding for components involved in both Hh and Wnt signaling are commonly altered in human BCCs [99,106]. It was shown that SuFu negatively regulates β-catenin signaling [107] and acts as a common regulator of Hh and Wnt signaling during *Xenopus* development [108]. Simultaneous inactivation of SuFu and Kif7 in adult epidermis results in the formation of BCCs that display increased nuclear β-catenin [109], reinforcing negative regulation of SuFu in β-catenin-dependent signaling. Moreover, overexpression of human GLI1 in frog epidermis induces BCC-like epidermal tumors which show specific upregulation of Wnt genes [96]. During epithelial transformation, Gli1 is able to induce the translocation of cytoplasmic β-catenin to the nucleus through modulation of E-cadherin [110]. Most recently, transcriptional profile of residual BCCs, which survive after treatment with Hh signaling inhibitor, reveals that Wnt signaling modulates cell identity of residual BCCs which may contribute to tumor relapse [111]. As a summary, in addition to the Hh pathway, dysregulation of Wnt/β-catenin signaling seems to be common during BCC development.

### 4.2. Squamous Cell Carcinoma (SCC)

SCC is recognized based on the tumor lesion composed of both proliferative basal cell and differentiated squamous cell layers. Epithelia arising from different parts of the body may develop different types of SCCs, including cutaneous SCC (cSCC), lung SCC (lSCC), and head and neck SCC (HNSCC). Each type of SCC has characteristics that can be distinguished by multidimensional genome-wide analyses [112]. One of the unique features for cSCC and HNSCC is their high degree of cellular heterogeneity [113,114,115]. As lSCC and HNSCC have been extensively discussed elsewhere [116,117,118], we mainly focus on cSCC in this review.

The incidence of cSCC is increasing remarkably each year due to a string of causative risk factors, including UV light from sun exposure, human papilloma virus (HPV) infection, chronic injury, arsenic exposure, immunosuppression, and inflammation [113,119]. Among these, UV light exposure is considered the most important accounting for 80–90% of identified cSCCs [120]. These prevailing factors promote the transformation of precancerous lesions, AK, to SCC in situ, invasive cSCC, and eventually metastatic SCC, as a consequence of multistep carcinogenesis. The malignancy of cSCC is favored by the accumulation of genetic and epigenetic alterations, viral pathogenesis, non-coding RNAs and dysregulation of signaling pathways [121]. The prognostic biomarkers of cSCCs can be determined by genetic, epigenetic, transcriptomic or proteomic analysis. These analyses provide invaluable information that hold the key for the selection of therapeutic intervention and for establishing comprehensive molecular landscapes of cSCC as future diagnostic biomarkers. The high rate of mortality and morbidity remains as a major concern attributed to the late diagnosis, ineffective treatment, relapse, and metastasis.

The main mutations of human cSCC are found in *TP53*, *CDKN2A*, *RAS*, *PTEN*, *EGFR*, and genes encoding for NOTCH receptors [121,122,123]. Around 75% of human cSCCs contain loss-of-function mutations in NOTCH1 or NOTCH2 [124]. In addition to the genes mentioned above, mutations of the telomerase reverse transcriptase (TERT) promoter are also found commonly in both human SCCs and BCCs [125,126]. Decades ago, in order to mimic SCC development in mice, a two-stage chemical carcinogenesis protocol, involving a single application of 7,12-Dimethylbenzanthracene (DMBA) followed by a repeated treatment with 12-O-Tetradecanoylphorbol-13-acetate (TPA), was exploited in murine models. DMBA administration causes the formation of DNA adducts, and successive treatment with TPA leads to sustained hyperplasia [127,128]. It is known that treatment with DMBA/TPA creates an accumulation of mutations in several critical genes, notably in *H-Ras* (A182T) [129], and provokes the progression of papilloma to malignant tumor, e.g., cSCC. Genetic alterations in genes encoding for RAS family members have been identified in a significant proportion of human tumors. More recently a genetic mouse model carrying oncogenic *K-Ras(G12D*) was widely utilized to study the process of tumorigenesis [130,131]. In adult epidermis, *K-Ras(G12D)* activation under the control of cell type-specific promoters allowed the origin for SCC formation to be defined. For instance, in combination with *Tp53* deletion, *K-Ras(G12D)* activation in IFE basal cells or HFSCs, but not in transient amplifying HF matrix cells, led to the development of SCCs [131]. Although transformation by oncogenic RAS is an important event, RAS mutations only account for approximately 8–20% of human SCCs [122,132]. Additional mutations in tumor suppressor genes, e.g., *TP53* or *TP63* [6,133,134,135], are required to drive the malignancy of SCCs. 

As previously described for BCCs, the cellular origin of SCCs affects the phenotype of the tumor. For instance, SCCs derived from the IFE are well-differentiated, whereas SCCs developing from the HFSCs frequently undergo EMT [136]. In addition to cell origin, the status of the originating cell also impacts on tumor formation. It has been shown that quiescent HFSCs are refractory to initiating tumors driven by activation of *K-RAS(G12D*) and *Tp53* depletion [137]. Other than gene mutations, dysregulation of signaling pathways has also been implicated in mouse skin tumorigenesis [138]. In mouse SCCs, dysregulation of the PI3K/AKT signaling pathway, known to regulate a wide-range cellular functions, such as cell proliferation and apoptosis [139,140], is commonly found in RAS-induced tumors derived from HFSCs [6].

Dysregulation of Wnt signaling, including Wnt/β-catenin and β-catenin-independent Wnt/Ca^2+^ signaling, emerges as a major cause of cSCC development and progression [61,122,141]. Genetic alterations in Wnt-related ligands, receptors or mediators were identified in human cSCCs using various genomic profiling approaches. The first comparative genomic hybridization analysis indicated amplification of chromosome arms 7q, 8q, 11q, and 17q, which containing *WNT* and *FZD* genes, in cSCCs [142]. This finding was reinforced by microarray analysis revealing that WNT5A and FZD6 are both upregulated as unique gene signatures in human cSCCs [141,143]. Recently, genomic profiling of 122 human cSCC samples identified clinically relevant genomic alterations (CRGAs) showing that the key mutations in cSCCs not only include truncations of *TP53* (85.2%), *CDKN2A* (61.5%), and *NOTCH1* (42.6%), but also alterations in Wnt-related genes, *LRP1B* (22.1%) and *APC* (8.2%) [122]. In addition, another genomic study of cSCC supports the previous genomic profiling studies in which Wnt signaling was one of the common mutated pathways in human cSCCs [123]. 

The potential role of Wnt antagonists, SFRPs and Dkks, in tumorigenesis has drawn interest. Several lines of studies indicated that SFRP1, which destabilizes β-catenin by interfering with Wnt-Fzd interactions, was downregulated in human SCC samples [141]. Accordingly, hypermethylation of several SFRP genes, including SFRP1, 2, 4, and 5, have been identified in cSCC tumors and recognized as critical prognostic determinants for cSCCs [144]. Although hypermethylated SFRP genes could have caused hyper-activation of Wnt signaling that might contribute to cSCC, mechanisms of how SFRPs modulate skin cancer pathogenesis will require further investigation. In addition to SFRPs, Dkk proteins, known as negative regulators of canonical Wnt signaling, could also potentially serve as bona fide tumor suppressors. Accompanied by elevated β-catenin expression levels, downregulation of Dkk1 was seen in human cSCCs [145]. A decrease in Dkk3 expression was also detected in cSCCs. Overexpression of Dkk3 in SCC cells significantly reduced proliferation and migration [146], suggesting that Dkk3 has inhibitory effects on SCC development.

**Figure 3:**
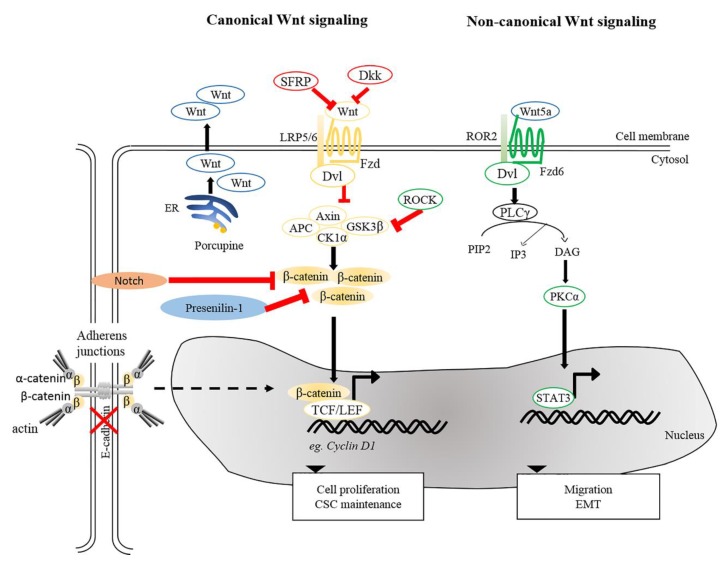
Wnt signaling pathways in cSCC. Canonical and non-canonical Wnt signaling participates in the maintenance of CSC, tumor progression, migration and EMT. Reduction of SFRPs and Dkks leads to activation of canonical Wnt signaling. Porcupine, an enzyme from the ER is needed for post-translational modification of Wnts to enable their transport and secretion. Other intricate factors, e.g., loss of E-cadherin or Presenillin-1, inhibition of Notch signaling and ROCK activation could modulate β-catenin signaling and activate genes involved in several cellular processes, including cell proliferation and CSC maintenance. For non-canonical Wnt signaling, interaction between Wnt5a and ROR2 facilitates EMT and invasive properties of cancer cells. Wnt5a is also required to activate PKCα and for STAT3 phosphorylation leading to tumorigenesis. ER, Endoplasmic reticulum; PKCα, protein kinase Cα; ROR2, receptor tyrosine kinase-like orphan receptor 2; STAT3, signal transducer and activator of transcription 3.

Aberrant accumulation of β-catenin protein or its presence in the nucleus in tumor cells is a common characteristic of SCCs [61,101,145,147,148]. Wnt/β-catenin signaling plays dual roles in both regulating normal SC self-renewal and maintenance and in cancer stem cells (CSCs). As discussed previously, depletion of β-catenin in carcinogen-induced SCCs halts tumor progression and eventually results in tumor regression, suggesting roles in maintenance of cutaneous CSCs [61]. This paradigm is in agreement with the idea that CSC-like properties in cSCC could be induced by aberrant expression of microRNA (miRNA) by activated canonical Wnt signaling [149]. Furthermore, it has been shown that tumor volume in a xenograft model of human cSCCs could be dampened upon β-catenin knockdown [62], underscoring the causal relationship between β-catenin and cell proliferation of cSCCs. These notions were based on a number of earlier studies showing that activation of β-catenin signaling, either by impairment of Notch [150], inactivation of Presenilin 1 [151] or activation of ROCK [152] caused an increase in the expression of cyclin D1 subsequently led to hyperproliferation (Figure 3). Conversely, aberrant expression of genes involved in cell cycle control can trigger the onset of skin tumorigenesis through activation of Wnt/β-catenin signaling. The cyclin-dependent kinase inhibitor 2A and 2B (CDNK2AB) locus, genes encoding for tumor suppressors p16 (INK4A), p14 (ARF), and p15 (INK4B) that inhibit cell cycle progression, is frequently lost in wide-range of tumors. A recent study indicated that loss of CDKN2ab allows development of SCC in the presence of active Wnt7b in a 129P2 mouse background [153]. Cell division cycle 20 (CDC20), another crucial cell cycle regulatory molecule, is commonly increased in cSCCs. Downregulation of CDC20 inhibits Wnt/β-catenin signaling, thereby suppressing the proliferation of cSCC cells and promoting apoptosis [154]. Taken together, these studies highlight the importance of Wnt signaling in regulating tumor cell proliferation and maintaining CSC phenotypes, both of which promote the SCC progression and aggressiveness.

Apart from β-catenin, TCF7L1 (also known as TCF3), a binding partner for β-catenin, is known to contribute to tumor prevalence and progression in the pathogenesis of several cancers [155,156]. Motif-based analysis of human and mouse tissues identified TCF7L1 as one of the transcription factors whose targets are prominently altered during the progression from normal skin to SCCs [157]. A recent study provided evidence for a tumor-promoting role of TCF7L1 in skin. Overexpression of TCF7L1 increased tumor incidence, promoted tumor growth, and enhanced migration independent of β-catenin [158]. In addition to TCF7L1, Wnt genes are also commonly dysregulated in diverse tumor cells, including prostate, ovarian, colon, and skin cancers [159,160,161,162]. Upregulation of Wnt5a is clearly observed in human cSCC. Wnt5a is of major importance in maintaining the tumor phenotype in human SCCs [143,162,163], implying that Wnt5a serves as an oncogenic driver in skin cancer. It was reported that oncogenic signals mediated by Wnt5a were acquired via activation of PKCα and STAT3 phosphorylation, but not through Wnt/β-catenin pathway [163]. As mentioned above, immunosuppressed organ transplant recipients are more prone to SCCs [164]. One plausible factor is the elevated risk of HPV infection among immunosuppressed populations as compared to the immunocompetent patients [165]. Interestingly, vaccination against HPV successfully reduces the incidence of SCC in immunocompetent patients [166]. One study demonstrated that the presence of HPV induces the development of skin carcinomas in mice upon exposure to UV or wounding [167]. In addition, transcriptome analysis of HPV-driven mouse cSCCs indicated that transcripts encoding for Wnt ligands and Porcupine, a protein required for Wnt secretion, were highly upregulated in cSCCs. Inhibition of Porcupine by a small molecule inhibitor reduced the initiation and progression of HPV-driven cSCC [168]. 

In addition to cSCC maintenance and progression, β-catenin has an impact on tumor metastasis via EMT, in which E-cadherin-bound β-catenin is released from cell membranes and an increased pool of cytoplasmic β-catenin may enter the nucleus to trigger β-catenin-dependent signaling [169,170,171]. This notion is supported by studies showing that suppression of E-cadherin expression accelerates SCC progression by increasing invasion [172,173]. Besides canonical Wnt signaling, non-canonical Wnt ligand and receptor, Wnt5a, and ROR2, were shown to be required for Snail-mediated EMT and invasive properties of cancer cells [162]. In agreement, upregulated Wnt5 at the leading edge of SCCs suggests the contribution of Wnt5a-dependent signaling to tissue invasion by SCCs [174]. Together, these analyses and findings provide insights into the contribution of Wnt signaling to cSCC pathogenesis.

## 5. Potential Therapeutic Targeting of Wnt Signaling

Targeting the Wnt signaling pathway is recognized as a potential therapeutic approach to treat various cancers. Currently, several preclinical and clinical trials on compounds targeting Wnt signaling are in progress. The conventional treatments for localized keratinocyte carcinomas include surgical excision, Mohs surgery, cryotherapy, curettage and electrodessication, topical treatment, photodynamic therapy, radiation therapy and chemotherapy individually or in combination [140,175,176,177]. For BCCs, surgery or topical application of Imiquimod is a first-line treatment for low risk superficial BCCs, leading to histological clearance in 80% of cases [178]. Patients with secondary BCCs also see benefits from topical Imiquimoid treatment [179,180]. As for high risk or metastatic BCCs, the Smo inhibitor Vismodegib, is more suitable for targeting the Hh signaling pathway with a 58% response rate among patients [175,181]. It has been shown that Vismodegib treatment caused tumor resurgence on BCC patients [182] which was likely due to the dormancy of residual BCCs. A recent study showed that dual inhibition of Hh and Wnt signaling by combination of Vismodegib and Wnt inhibitor (anti-Lrp6 antibody) reduced the residual BCC burden and facilitated differentiation [111]. Another study indicated that the tumor relapse was attributed to the dormancy of Lgr5^+^ cells and revealed that targeting Lgr5^+^ cells by inhibition of Wnt signaling (Wnt inhibitors, Lgk974) in combination with Vismodegib treatment resulted in regression of BCCs [183]. This opens the door to future possibilities of clinical trials focusing on the cross-interaction between signaling pathways, e.g., Hh and Wnt signaling. 

For cSCCs patients, the primary treatments are surgical excision, Mohs surgery and/or adjuvant radiation therapy. Radiation therapy followed by adjuvant chemotherapy is recommended for metastatic cSCCs [184,185]. The current therapeutic strategies, such as surgical excision and topical treatment, are sufficient for non-aggressive keratinocyte carcinomas. However, patients developing metastatic BCCs or cSCCs, have a poor outcome on current therapeutic treatments. Given the importance of Wnt signaling in driving cSCC development, Wnt antagonists and Wnt-related mediators mentioned above are potential therapeutic targets to treat cSCCs (Table 1). Porcupine is an enzyme which facilitates the posttranslational acylation of Wnt and consequently leads to secretion of Wnt ligands [186]. Thus, it is conceivable that inhibition of Porcupine will hamper the secretion of Wnt ligands, which in turn would inhibit activation of Wnt signaling. A number of potent Porcupine inhibitors have been developed, including Wnt-c59, IWP, LGK974, and ETC-159, and some are currently in phase I/II clinical trials. As an example, LGK974 was exploited to target Wnt-driven human HNSCC [187], as well as to impair HPV-driven cSCC initiation and progression [168]. Another potential Wnt signaling target is Tankyrase, an enzyme that is crucial component for the synthesis of Axin. Several small molecule inhibitors targeting Tankyrase, e.g., IWR and XAV939, have been designed to stabilize the production of Axin, which facilitates the degradation of β-catenin and eventually reduces activation of Wnt/β-catenin signaling [188,189]. In addition, aberrant expression of β-catenin is considered as an indicator of malignancies. Targeting β-catenin signaling may be of interest as it potentially eliminates CSCs and consequently eradicates SCCs. Hence, small molecule inhibitors of β-catenin, such as ICG-001 and PRI-724, have been developed in order to interfere with the recruitment of β-catenin by its coactivator, CREB-binding protein (CBP) [190]. Moreover, Wnt antagonists, such as Dkks, could be potential targets given their involvement in modulating skin cancer pathogenesis. Increasing the secretion of Dkks, e.g., Dkk3, could be an option due to their tumor suppressor roles as mentioned above. A preclinical study reported that adenoviral vector expressing Dkk3 reduced the growth of tumors by inducing apoptosis in a prostate cancer model [191], and currently this approach is in phase I/II clinical trials (National Clinical Trial (NCT) 01931046). 

Despite of the advanced treatments available for cSCCs, these treatments may gradually lose durable responses or cause a number of side effects, and tumors may relapse or become resistant to treatment [192]. Also, there are limited therapeutic approaches and sparse information about systemic therapies in combating metastatic cSCCs according to the latest National Comprehensive Cancer Network guidelines. Systemic therapies, including chemotherapy, immunotherapy, hormone therapy, and targeted drugs, alone or in combination have been used for cSCC clearance. However, it was reported that the recurrence rate was higher than in the combination regimen than single agent therapy [193]. In addition, a phase III clinical trial revealed that the combination of chemotherapy with targeted drugs improved progression-free survival, but not the overall survival in SCC patients [194].

Owing to the involvement of Wnt signaling in diverse cellular functions across different normal tissues, treatments targeting Wnt signaling might affect healthy tissues or organs. For instance, porcupine inhibitors, e.g., LGK974 and Wnt-c59, might negatively impact on bone volume and strength [195]. It was also shown that inhibition of Tankyrase activity might perturb bone homeostasis and cause bone loss [196]. Nevertheless, strategies interfering with β-catenin-CBP interaction or inducing DKK3 expression so far have no reported detrimental effects. It is thus worth further investigating the exploitation of Wnt antagonists as future cSCC interventions. In summary, the development of inhibitors targeting oncogenic Wnt signaling essential for tumor initiation and progression is a potential treatment for keratinocyte carcinomas.

## 6. Conclusions

In this review, we emphasize the crucial roles of Wnt signaling in different aspects of keratinocyte carcinoma development, including their participation in tumor initiation, progression, invasion, and metastasis. Canonical Wnt signaling is essential for keratinocyte carcinoma initiation and progression by enhancing tumor cell proliferation and regulating the maintenance of CSCs. On the other hand, non-canonical Wnt signaling is necessary for regulating the process of tumor invasion and metastasis. Despite the availability of various analyses to screen the mutations or biomarkers for these cancers, it is plausible that due to heterogeneity some tumors might escape detection due to factors from the tumor microenvironment and existing quiescent CSC populations. Future investigations should focus not only on the intricate molecular communications within tumors, but also on extrinsic factors from stromal cells, including fibroblasts and immune cells, as well as identification of CSCs. In view of the complexity of multiple signaling coordinating skin tumorigenesis, it will be of utmost importance to better understand and dissect the cross-interactions of Wnt signaling with other oncogenic pathways. This would allow the development of new effective therapeutic interventions to eradicate Wnt signaling-related malignancies.

## Figures and Tables

**Table 1:** Potential Inhibitors Targeting the Wnt Signaling Pathway in Preclinical and Clinical Trials.

Compound	Target	Clinical Trial Number	Trial Phase	Reference
Lgk974	Porcupine	NCT01351103 NCT02278133	I I	[168]
IWP	Porcupine	-	Preclinical	
Wnt-c59	Porcupine	-	Preclinical	[197]
ETC-159	Porcupine	NCT02521844	I	
XAV939	Tankyrase	-	Preclinical	[198]
IWR	Tankyrase	-	-	[188]
ICG-001	β-catenin-CBP interaction	-	Preclinical	[199]
PRI-724	β-catenin-CBP interaction	NCT 01606579 NCT 01302405	I/II	[200]
E7386	β-catenin-CBP interaction	NCT03833700 NCT03264664	I	
